# Role of telehealth in outbreaks—Where the classical healthcare systems fail

**DOI:** 10.1017/ice.2020.120

**Published:** 2020-04-13

**Authors:** Ata Mahmoodpoor, Mohammad Amin Akbarzadeh, Sarvin Sanaie, Mohammad-Salar Hosseini

**Affiliations:** 1 Department of Anesthesiology and Critical Care Medicine, Faculty of Medicine, Tabriz University of Medical Sciences, Tabriz, Iran; 2 Student Research Committee, Tabriz University of Medical Sciences, Tabriz, Iran; 3 Aging Research Institute, Tabriz University of Medical Sciences, Tabriz, Iran; 4 Research Center for Evidence-Based Medicine, Tabriz University of Medical Sciences, Tabriz, Iran

*To the Editor*—Outbreaks impose massive burdens on healthcare systems. For example, >510 deaths were reported among the healthcare workers (HCWs) during the last Ebola outbreak, and by February 24, 2020, >3,300 HCWs had been infected with SARS-Cov-2 in China alone.^1,2^ This issue represents the essence of outbreaks—a mass of patients who require prompt medical care. Under these conditions, the classical healthcare system cannot manage the large number of COVID-19 patients immediately and effectively.^3^

Telehealth provides vital services through the application of information and communication technologies for each stage of managing diseases, research, and continuing education.^4^ With telecommunication, visits are more economical and preserve government, community, and family resources, which are already limited during global outbreaks. It can be used to provide rapid diagnosis, and it enables caregivers to act quickly.^5^ With the appropriate utilization of telecommunication, it is possible bring access to medicine and concurrent therapy in hard-to-reach regions and communities, facilitating direct-to-patient or specialty consultation services from a distance, which is cost-effective and improves the efficacy of follow-up.^6^

**Fig. 1.**
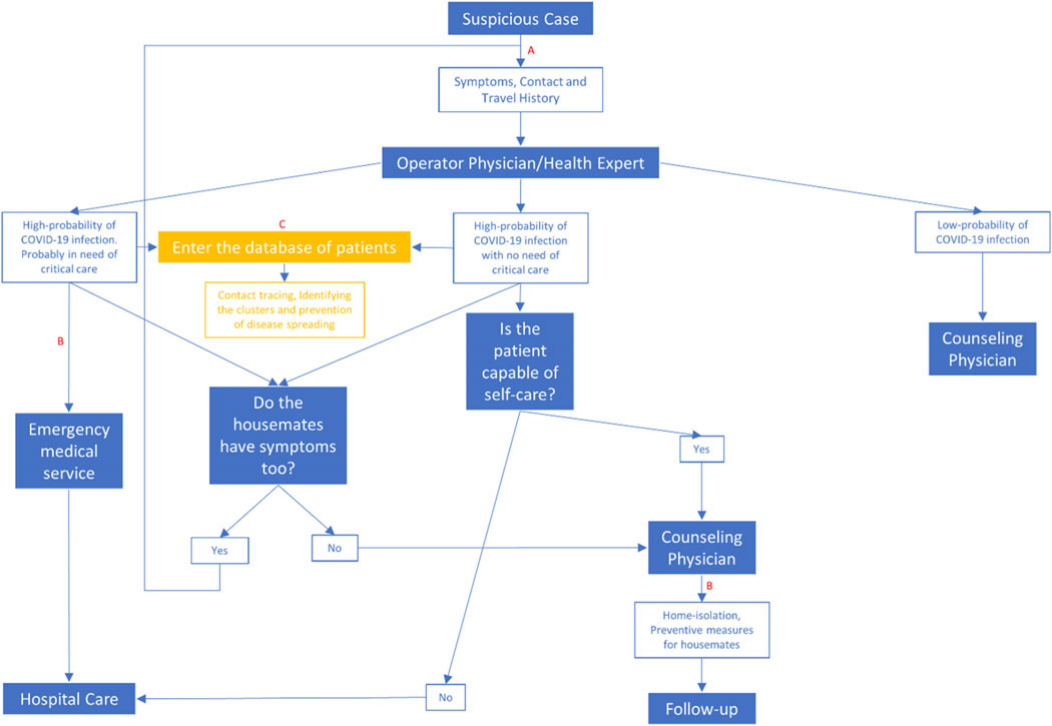
Algorithm of telemedicine application during the COVID-19 outbreak. This model optimizes the use of telehealth, telemedicine, and emergency medical services. This model obviates unnecessary referrals, reduces the load of the healthcare system, and systematizes hospital care. Axis ‘A’ displays the first application of telemedicine, which omits unnecessary referrals by implementing a diagnostic role. Axis ‘B’ provides the required care under the supervision of a physician without the need for an in-person visit. Axis ‘C’ contributes to limiting the outbreak and social management in the outbreak setting.

The benefits of implementing telehealth in outbreaks comprise 8 main areas of focus:

1. The available admission capacity and the number of HCWs are limited, and the hospital infrastructure may not be adequate to serve all patients. Figure 1 presents an optimum model of telemedicine that could aid medical and social management during an outbreak.
2. The more patients referred to healthcare facilities, the more HCWs are at risk of being infected. Also, most referrals are unnecessary and merely increase the load on the healthcare system. Furthermore, people referred to hospitals are at an incredibly higher risk of infection. More than 40% of the infections are assumed to be hospital related, though they should be quite simple to prevent (Fig. 1).^7^
3. In affected regions, most healthcare facilities are dedicated to the management of COVID-19 patients. Therefore, noncritical patients (like patients with chronic and metabolic disorders such as hypertension, diabetes, and hyperlipidemia) have a lower priority. As a result of self-isolating, these people, mostly the older adults, cannot attend the healthcare units. A remote follow up and visit of these patients could prevent the consequences of poorly controlling the diseases.
4. During a worldwide health emergency, some people with less mobility (ie, disabled patients) may not even be able to access the nearest local care center. Telehealth can facilitate assistance in their location while simultaneously providing clinicians instant access to their medical records.
5. In many countries, medical schools and universities are among the closed establishments. The duration of this shutdown is unknown, and medical education should be continued through the online and virtual classes. Medical conferences and gatherings could be scheduled likewise.
6. Proper use of popular social networks could significantly aid in prevention by effectively educating the populace.
7. Using a referral system based on telehealth can provide immediate access to medical records of infected patients. These records could be analyzed to identify the cluster “hot spots.”^8^
8. Developing a network could help clinicians discuss special cases and share the latest findings and their evidence-based experiences regarding the daily advances and findings related to the disease. The network provides an advantage during health crises by enabling rapid and efficient responses.

However, adopting telemedicine or telehealth may be difficult because of costs, the challenges of high technology, lack of awareness, limited access to high-speed internet, reimbursement problems, and availability of technical support are the issues that may slow down the application of telehealth.^9^ These barriers are present in both developing and developed countries. In developing countries, the primary obstacle is most likely the implementation of new technology (eg, high-speed internet), and in developed countries, the most concerning matter is the need for new behavioral patterns and how to develop them. The essentials are easy to implement, and with advantages such as improved investigations, disease control, clinical case management, and information enhancement, societies can benefit from the telehealth method. In outbreaks, it is important to rapidly increase treatment units, to expand their reach through telehealth, and to develop and disseminate appropriate treatment methods for communities, and a great demand for support and assistance is expected. Such interventions using telehealth could eventually connect all healthcare personnel across the world, which could be very beneficial during a pandemic. At this point, telehealth and telemedicine comprise one of the few reliable strategies that could intervene when the classical system of healthcare becomes ineffective.
